# Impact of Tumour Segmentation Accuracy on Efficacy of Quantitative MRI Biomarkers of Radiotherapy Outcome in Brain Metastasis

**DOI:** 10.3390/cancers14205133

**Published:** 2022-10-20

**Authors:** Seyed Ali Jalalifar, Hany Soliman, Arjun Sahgal, Ali Sadeghi-Naini

**Affiliations:** 1 Department of Electrical Engineering and Computer Science, Lassonde School of Engineering, York University, Toronto, ON M3J 1P3, Canada; 2 Department of Radiation Oncology, Odette Cancer Centre, Sunnybrook Health Sciences Centre, Toronto, ON M4Y 3M5, Canada; 3 Department of Radiation Oncology, University of Toronto, Toronto, ON M5G 1V7, Canada; 4 Physical Sciences Platform, Sunnybrook Research Institute, Sunnybrook Health Sciences Centre, Toronto, ON M4Y 3M5, Canada

**Keywords:** deep learning, therapy outcome prediction, radiotherapy, brain metastasis, radiomics, segmentation

## Abstract

**Simple Summary:**

Radiotherapy is a major treatment option for patients with brain metastasis. However, response to radiotherapy is highly varied among the patients, and it may take months before the response of brain metastasis to radiotherapy is apparent on standard follow-up imaging. This is not desirable, especially given the fact that patients diagnosed with brain metastasis suffer from a short median survival. Recent studies have shown the high potential of machine learning methods for analyzing quantitative imaging features (biomarkers) to predict the response of brain metastasis before or early after radiotherapy. However, these methods require manual delineation of individual tumours on imaging that is tedious and time-consuming, hindering further development and widespread application of these techniques. Here, we investigated the impact of using less accurate but automatically generated tumour outlines on the efficacy of the derived imaging biomarkers for radiotherapy response prediction. Our findings demonstrate that while the effect of tumour delineation accuracy is considerable for automatic contours with low accuracy, imaging biomarkers and prediction models are rather robust to imperfections in the produced tumour masks. The results of this study open the avenue to utilizing automatically generated tumour contours for discovering imaging biomarkers without sacrificing their accuracy.

**Abstract:**

Significantly affecting patients’ clinical course and quality of life, a growing number of cancer cases are diagnosed with brain metastasis (BM) annually. Stereotactic radiotherapy is now a major treatment option for patients with BM. However, it may take months before the local response of BM to stereotactic radiation treatment is apparent on standard follow-up imaging. While machine learning in conjunction with radiomics has shown great promise in predicting the local response of BM before or early after radiotherapy, further development and widespread application of such techniques has been hindered by their dependency on manual tumour delineation. In this study, we explored the impact of using less-accurate automatically generated segmentation masks on the efficacy of radiomic features for radiotherapy outcome prediction in BM. The findings of this study demonstrate that while the effect of tumour delineation accuracy is substantial for segmentation models with lower dice scores (dice score ≤ 0.85), radiomic features and prediction models are rather resilient to imperfections in the produced tumour masks. Specifically, the selected radiomic features (six shared features out of seven) and performance of the prediction model (accuracy of 80% versus 80%, AUC of 0.81 versus 0.78) were fairly similar for the ground-truth and automatically generated segmentation masks, with dice scores close to 0.90. The positive outcome of this work paves the way for adopting high-throughput automatically generated tumour masks for discovering diagnostic and prognostic imaging biomarkers in BM without sacrificing accuracy.

## 1. Introduction

While the exact incidence of brain metastasis (BM) is difficult to determine, about 70,000 to 400,000 new cases of BM are annually diagnosed in the United States alone [1]. The incidence of BM appears to be ten times higher than the incidence of primary malignant brain tumours [2]. Because of the accompanying neurologic symptoms, psychological effects, and changes in oncologic treatment plans, the development of BM may significantly affect a patient’s clinical course [3].

Radiation therapy, chemotherapy, immunotherapy, and surgery are the main treatment options for the management of metastatic brain tumours. Because systemic therapy often fails to penetrate the blood–brain barrier, local brain-directed therapies such as radiation or neurosurgical resection are commonly used [4], although there has been some progress in the use of systemic targeted therapy for the management of patients with BM [5]. Whole-brain radiation therapy (WBRT), hypofractionated stereotactic radiotherapy (SRT), and single-fraction stereotactic radiosurgery (SRS) are available options for radiotherapy. Apart from these options, recent studies have shown that a combination of immunotherapy with radiotherapy is associated with improved overall survival compared to radiotherapy alone in patients with brain metastasis who received surgery for their primary cancer [6]. Specially, immunotherapy has shown significant control of intracranial metastasis in patients with melanoma [6]. Moreover, a combination of immunotherapy, e.g., immune checkpoint inhibitors, with radiation therapy has shown promise for the treatment of brain metastasis in non-small-cell lung cancer [7,8]. Magnetic resonance imaging (MRI) is the primary imaging modality used for diagnosis, treatment planning, and treatment outcome assessment in BM. The conventional treatment planning and outcome evaluation process includes obtaining MRI scans before (baseline) and after radiation therapy, during several follow-up sessions. This procedure involves precise delineation of the tumour, which is often carried out by experienced radiation oncologists and neuroradiologists. The response assessment in neuro-oncology brain metastasis (RANO-BM) group [9] has developed standard criteria for evaluating radiotherapy outcome in BM using serial MRI. The local response of BM to stereotactic radiation therapy is determined by changes in tumour size on follow-up serial imaging [9] and may be classified into two categories: local control (LC; shrinking or stable tumour) and local failure (LF; enlarging tumour excluding adverse radiation effect). In case of immunotherapy, the immunotherapy response assessment in neuro-oncology (iRANO), a modification of RANO criteria, is used for response assessment [10].

Since the conventional response assessment is based on the changes in tumour size following treatment, it may take months before a local response is apparent on standard follow-up images. This is not desirable, especially given the fact that patients diagnosed with BM suffer from a short median survival. To make things more complicated, lesion enlargement after treatment on MRI is not necessarily a sign of tumour progression, but also of a condition known as pseudoprogression due to adverse radiation effect [11].

To mitigate these complications, and since the local response is highly varied even among the patients going through the same treatment regimen (because of the patient and/or tumour-related factors), there have been efforts to develop a tailored treatment strategy based on the patient’s subgroup and predicted survival [12]. Early efforts involved stratifying patients based on factors such as age, performance status, control of primary tumour, and extent of extracranial disease using recursive partitioning analysis (RPA) [13,14,15], followed by more complicated stratification methods such as diagnosis-specific graded prognostic assessment (DS-GPA) [12,16]. Significant prognostic factors such as the primary site of cancer, age, and Karnofsky performance status (a score of 0–100 to quantify a patient’s ability to perform daily activities) are used to define the DS-GPA prognostic index. A GPA of 4.0 correlates with the best prognosis, whereas a GPA of 0.0 corresponds to the worst prognosis.

The successful application of artificial intelligence (AI) for diagnostic purposes has led to the development of AI-based cancer imaging analysis, which is now being employed to meet more sophisticated therapeutic requirements, such as patient stratification and therapy outcome prediction. Neuroimaging data can be used to extract quantitative and semiquantitative image features that are often beyond human vision. Given the abundance of MRI and CT data acquired as part of the standard of care for patients with BM, there is a huge amount of data available for mining useful prognostic features to help with response prediction before or early after therapy. Radiomics has been introduced as a formal approach for extracting and discovering quantitative diagnostic and prognostic features from medical images [17]. The quantitative features extracted from the medical imaging data for biomarker discovery in radiomic analysis are usually categorized as [18] morphological features that quantify the geometry and shape of the region of interest such as sphericity, first-order statistical features that describe the voxel intensities without considering the spatial relationship between them such as intensity mean or standard deviation, second-order texture features that are obtained by calculating the statistical interrelationships between the intensity of neighbouring voxels, such as those based on grey-level co-occurrence matrix (GLCM), and higher-order statistical features that are obtained after applying a transformation on the image, e.g., features extracted from textural parametric images.

New studies have shown connections between tumour radiomic signatures and their phenotypes, genomic, and proteomic profiles [19,20,21,22]. Inspired by such connections, several studies have explored the potential of radiomic features in conjunction with machine learning (ML) to develop an efficient and noninvasive method of characterizing metastatic brain tumours and predicting their treatment outcome. Karami et al. [23] have proposed an MRI-based radiomic framework for early prediction of treatment outcome in patients with BM treated with hypofractionated stereotactic radiation therapy (SRT). The proposed quantitative MRI (qMRI) biomarkers were developed through a multistep feature extraction/reduction/selection framework and fed to a support vector machine (SVM) classifier to predict the radiotherapy outcome in terms of LC/LF. A recent study by Mouraviev et al. has investigated whether MRI radiomic features provide any additional value to clinical variables for predicting local control in BM following SRS [24]. The results show that the addition of radiomic features to the clinical variables increases the area under the ROC curve considerably.

In quantitative cancer imaging and radiomics, the entire tumour volume should be segmented to determine the region of interest (ROI) for analysis. The segmented tumour boundaries also determine other relevant regions such as the peritumoural areas for further analysis. Therefore, accurate tumour delineation is a fundamental step in oncologic radiomics. Manual segmentation of tumour on volumetric images acquired at several imaging sessions for each patient is a tedious and time-consuming job. Automatic segmentation of tumours facilitates radiomic analyses and streamlines the standard process of therapy outcome evaluation in clinic considerably, possibly at the cost of less accuracy.

In this paper, we evaluate the impact of tumour segmentation accuracy on efficacy of the quantitative MRI biomarkers for radiotherapy outcome prediction in BM. A cascaded attention-guided framework is proposed to accurately segment the tumour on the baseline and first follow-up automatically. Using the segmentation masks generated with this framework, we extracted radiomic biomarkers associated with the tumour and peritumoural areas from T1-weighted (T1w) and T2-weighted fluid attenuation inversion recovery (T2-FLAIR) MR images to develop predictive models of radiotherapy outcome. The study results show that to extract meaningful and distinguishing biomarkers, tumour masks should be reasonably accurate but not necessarily matching completely the ground-truth identified manually by expert clinicians. In particular, the results of outcome prediction using the ground-truth and the automatically generated masks are comparable with the top selected biomarkers shared between the two approaches.

## 2. Materials and Methods

### 2.1. Data Acquisition

This research was carried out in compliance with the institutional research ethics board approval from Sunnybrook Health Sciences Centre, Toronto, ON, Canada. The imaging data were obtained from 124 patients who had been diagnosed with BM and were treated with hypofractionated SRT over five fractions. Gadolinium contrast-enhanced T1w and T2-FLAIR images were acquired before the treatment (baseline) and at the first follow-up after SRT. Both the T1w and the T2-FLAIR had an in-plane resolution of 0.5 mm. The slice thickness for the T1w and T2-FLAIR images was 1.5 mm and 5 mm, respectively. T2-FLAIR images were coregistered on the T1w images, and both images were resampled to make the voxel size isotropic ($0.5\times 0.5\times 0.5\;{\mathrm{mm}}^{3}$), rendering the size of both MRI volumes at 512×512×348 voxels. The voxel intensities in each image were normalized between 0 and 1. The treatment-planning tumour outlines delineated by expert oncologists and neuroradiologists for each patient were included in the dataset. Among 124 patients (156 lesions), 99 patients (116 lesions) were randomly assigned for training and optimization of the predictive models (10 patients with 15 lesions as the validation set for optimizing the model hyperparameters) and 25 patients (40 lesions) were kept unseen as an independent test set.

After SRT, the patients underwent follow-up MRI scans every two to three months. The lesions were monitored using serial MRI, and a radiation oncologist and a neuroradiologist determined the local response for each lesion using the RANO-BM [9] criteria. The local outcome was categorized as either LC (complete response, partial response, or stable disease) or LF (progressive disease) based on the response determined in the last follow-up session. Using serial imaging (including perfusion MRI) and/or histological confirmation, the adverse radiation effect (ARE) was diagnosed and distinguished from local progression [25], based on the report by Sneed et al. [11]. Following these criteria, a total of 93 and 63 lesions were classified as LC and LF, respectively.

### 2.2. System Overview

**Figure 1:**
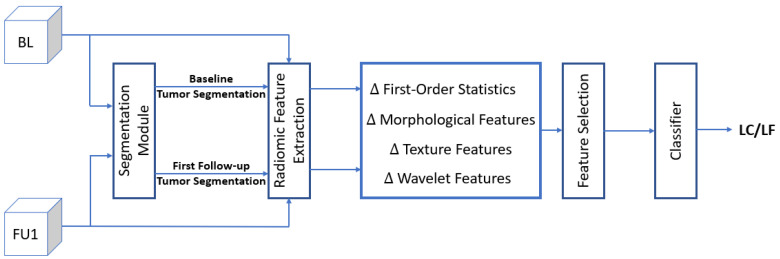
The overall system for SRT outcome prediction. The segmentation module segments tumours on the baseline (BL) and the first follow-up (FU1) MR images. Using the MR images and segmentation masks, different radiomic features are extracted. The features are then sorted based on their relevance/redundancy and a subset of them identified through feature selection are used to train a classifier for predicting the local outcome.

Figure 1 shows the overall framework adapted for predicting the local outcome in patients diagnosed with BM. Since a tumour segmentation mask is necessary for extracting radiomic features, in this study we replaced the manual tumour masks with the ones generated automatically by our proposed segmentation framework. Using the MR images of both modalities (T1w and T2-FLAIR) and the automatically generated segmentation masks, the radiomic features are extracted from the tumour and peritumoural regions for both the baseline and first follow-up sessions. The change in each feature is then calculated at the first follow-up relative to the baseline. The number of features is reduced using feature selection techniques. The selected features are then fed to train a classifier to predict the therapy outcome. More details on different components of the framework are provided in the following subsections.

### 2.3. Segmentation Module

**Figure 2:**
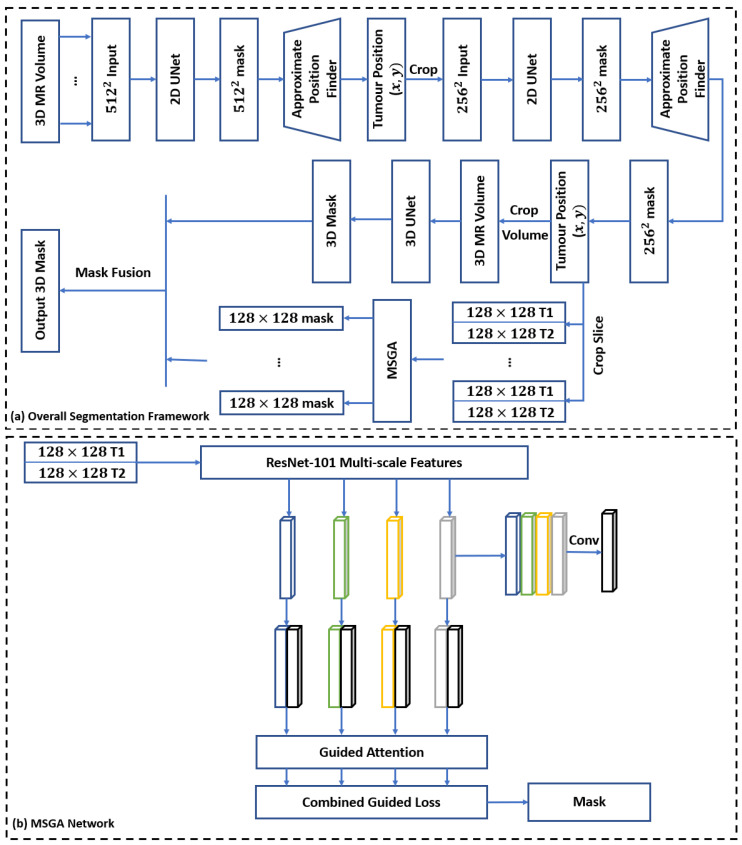
(**a**) Segmentation framework overview. For a volumetric input image (contrast-enhanced T1-weighted, 512×512×128 voxels), first, all slices are fed to a 2D UNet one by one. The generated masks from the 2D UNet are used to find an approximate tumour position (x,y). The volumetric input image is then cropped around (x,y) into a 256×256×128 voxel volume. A similar procedure is performed to reduce the size of the volumetric image containing the tumour to 128×128×128 voxels. This volume is then fed into a 3D UNet for segmentation with no patching. The slices of this volume are also fed to MSGA, after concatenation with the coregistered T2-FLAIR image, and the segmentation masks of MSGA are then fused with those of 3D UNet. (**b**) The MSGA network structure. Features extracted at different scales (shown in blue, green, orange, and gray) from ResNet-101 are concatenated and convolved (shown in back) and then self-concatenated and fed into the guided attention module. The resulting self-guided features are fed into the guided loss.

Figure 2 shows the proposed framework for automatic tumour segmentation of brain tumours on MRI. Two cascaded 2D UNets [26] are responsible to find the approximate position of the tumour. The image is cropped around the tumour after determining its approximate position to reduce the size of the input image for the next network. More specifically, the input size of the first and second 2D UNets are 512×512 and 256×256 pixels, respectively, while the input size of the subsequent 3D UNet [27] is 128×128×128 voxels. The cropping is done to limit the scope of input to the areas that potentially include tumours and eliminate irrelevant parts to obviate the need for patching or resizing the input of the 3D UNet and multiscale self-guided attention (MSGA) [28] networks due to their memory limitations. Patching the input volume results in the loss of contextual information (for example, a tumour may split apart in several patches), whereas resizing it causes the loss of precise local information. Using the approximate position of the tumour determined with the cascaded 2D UNets to crop the input volume around the tumour region, it would be feasible to preserve both the local and contextual information. For finding the approximate tumour position using the associated 2D segmentation masks, a logical OR operation is applied to all the masks to generate a single mask displaying an upper bound of the tumour areas in different slices. The connected components in the single mask are then identified, and the centre of each connected component is considered to be the approximate centre of the corresponding tumour. The approximated centres are used to crop the image around the tumour region. In case of multiple tumour presence in an input image, the tumours are treated separately. The final output mask is generated by thresholding the averaged probability maps of the output of 3D UNet and MSGA networks with a threshold level of 0.5.

The suggested architecture combines the 2D UNet, 3D UNet, and MSGA networks in order to integrate their advantages while minimizing their limitations. The 2D UNet architecture’s good performance in various segmentation tasks is due to its ability to collect context and enable localization by combining a contracting path and a symmetric expanding path with skip connections in between. However, a 2D UNet does not consider the 3D spatial dependencies between the voxels during segmentation. The 3D UNet takes into account such spatial dependencies at the cost of higher memory consumption, while the MSGA network mitigates the limitation of 3D UNet in capturing long-range dependencies. Particularly, the MSGA network enables capturing richer contextual dependencies and neglecting irrelevant information using an embedded mechanism of attention. Moreover, the utilization of interdependent channel maps in MSGA, which enables the network to integrate local features with their corresponding global dependencies, makes it efficient in our application, where the network is fed with two channels of T1-weighted and T2-FLAIR images.

The segmentation framework was only trained on the images of the training set acquired at the baseline, while those training images acquired at the first follow-up and all images of the test set were kept unseen to the framework.

### 2.4. Radiomic Feature Extraction

Using the radiomic features, a quantitative description of each tumour can be derived from imaging data. A total of 3436 radiomic features were extracted from the tumour region and its 5 mm margin [23] on T1w and T2-FLAIR MR volumes acquired at baseline and the first follow-up using the automatically generated masks. The extracted radiomic features included the first-order statistics (including energy, entropy, etc., a total of 19 features), 2D and 3D morphological features (including maximum 2D diameter, flatness, sphericity, a total of 26 features), and texture features derived based on the gray level co-occurrence matrix (GLCM) (total of 24 features), gray level run length matrix (GLRLM) (total of 16 features), gray level size zone matrix (GLSZM) (total of 16 features), neighbouring gray tone difference matrix (NGTDM) (total of 5 features), and gray level dependence matrix (GLDM) (total of 14 features). All features were extracted using the pyradiomics package in Python [29]. The morphological features were derived from the generated binary masks. Other features were extracted from both the original MR images and the associated wavelet-filtered images. For the latter, low- and high-pass wavelet filters were applied in the x, y, and z directions of the 3D images resulting in eight filtered images of I_HHH_, I_HHL_, …, I_LLL_ for each original image. The delta radiomic features were calculated using the relative difference between the value of each feature at baseline and the first follow-up.

### 2.5. Feature Selection

In machine learning, feature selection approaches allow to save computation time, improve prediction performance, and gain a deeper knowledge of the data [30]. The delta radiomic features were processed through a feature selection procedure using the minimum redundancy maximum relevance (mRMR) method [31] with a mutual information quotient (MIQ) criterion. The mRMR method tends to select a feature subset with high correlation to the target class (output) and low interfeature correlations. The F-statistics were used to calculate the correlation with the target class (relevance), while the Pearson correlation coefficient was applied to calculate the interfeature correlations (redundancy). The MIQ score represents the quotient of relevance and redundancy. Ultimately, among the 3436 features, seven features were selected using the mRMR technique. For the feature selection process, only the training data was applied to prevent data leakage from the training set to the test sets.

### 2.6. Classifier

A support vector machine (SVM) with a Gaussian kernel was adapted as the classifier for outcome prediction because of its demonstrated performance, versatility coming from different kernels, and effectiveness in high-dimensional space [32]. The selected delta radiomic features in the training set were normalized between 0 and 1 and used to train the SVM classifier with various hyperparameters. The best SVM model was selected based on the performance of the model on the validation set using the area under the receiver operating characteristics (ROC) curve (AUC) criterion. In this model, the penalty (C), gamma, and the tolerance parameters were set to 1, $\frac{1}{num\_features}\;$, and 1e−3, respectively. A class weight inversely proportional to the class frequencies was also assigned to each class. Finally, the model was tested on the independent test set, where the corresponding features were normalized using the normalization coefficients obtained for the training set.

## 3. Results

Table 1 demonstrates the results obtained with the proposed framework on the training and test sets for tumour segmentation at the baseline and first follow-up in terms of dice similarity coefficient (DSC), Hausdorff distance (HD), and volume estimation error (VEE). For comparison, the segmentation results are also reported for the cascaded 2D UNets, 3D UNet, and the cascaded 2D and 3D UNets. The results of Table 1 demonstrate the superiority of the proposed framework compared to the other models in accurately segmenting tumours on the baseline and first follow-up, which later demonstrates its importance in predicting the local response to radiotherapy.

Table 2 shows the most important features selected using the mRMR feature selection method for different segmentation models. A comparison between the selected features extracted using the ground-truth masks and the cascaded 2D and 3D UNets + MSGA model shows that six out of seven features are shared between the two models. This indicates that the tumour masks generated using the proposed segmentation framework may be sufficiently accurate for MRI radiomic analysis in this application. The number of shared features with the ground-truth gradually reduces when the accuracy of segmentation models decreases, with no shared feature for the cascaded 2D UNets.

**Figure 3:**
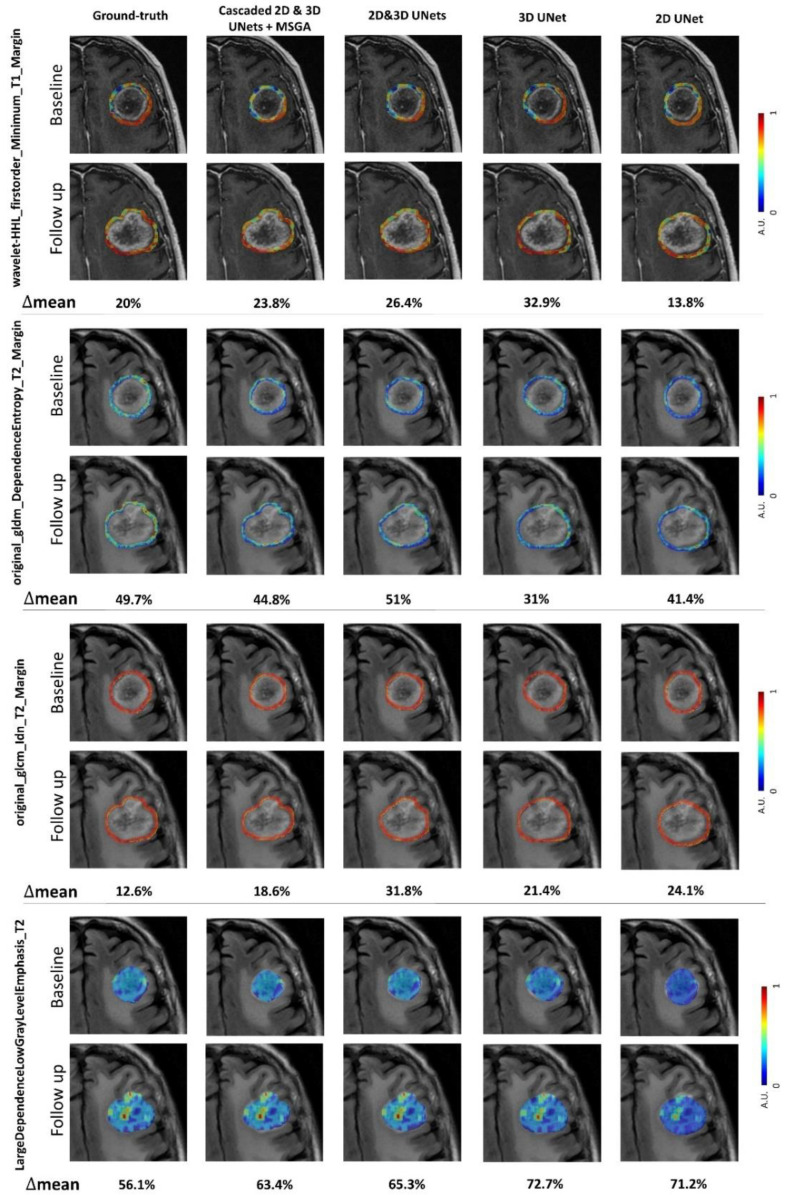
Representative parametric maps of the top four selected features based on the ground-truth (Table 2) overlayed on the associated MR images acquired at the baseline and the first follow-up from a representative tumour with an LF outcome. The parametric maps are shown for the ground-truth mask and the segmentation masks generated automatically using different models. Δmean for each feature is the mean relative change from the baseline at the first follow-up that is calculated for different masks.

Figure 3 demonstrates the parametric maps of the top four selected features based on the ground-truth (Table 2) overlayed on the baseline and follow-up images of a representative tumour with an LF outcome. The parametric maps are shown for the ground-truth mask and the automatically generated segmentation masks obtained from different models. The figure demonstrates that the parametric maps and the relative changes of features from the baseline are in good agreement with the ground-truth for the models with acceptable segmentation accuracy.

Table 3 shows the results of outcome prediction on the independent test set in terms of accuracy, sensitivity, specificity, AUC, and F1-score, using the selected radiomic features associated with different tumour segmentation models. The prediction performance of the radiomic model associated with the tumour masks generated automatically using the cascaded 2D and 3D UNets + MSGA network is reasonably close to the ground-truth model (AUC of 0.78 versus 0.81). On the other hand, the tumour masks generated using the cascaded 2D UNets or 3D UNet have resulted in considerably lower performances of the corresponding radiomic models (AUC of 0.62 and 0.67, respectively) compared to the ground-truth. The sensitivity and specificity of the ground-truth model versus the model developed with the cascaded 2D and 3D UNets + MSGA network are 83% and 78% versus 77% and 83%. This also indicates reasonably close performance of the ground-truth masks and those generated automatically by the proposed segmentation framework in radiomic modeling for treatment outcome prediction. The sensitivity of the ground-truth model is slightly better which shows it can detect local failure cases more accurately. On the other hand, the model developed with the cascaded 2D and 3D UNets + MSGA network performs better in detecting the local control cases. These observations imply that although accurate segmentation of tumour region is important in developing radiomic-based predictive models of therapy response, such models can be reasonably robust to imperfections in their input tumour masks.

**Figure 4:**
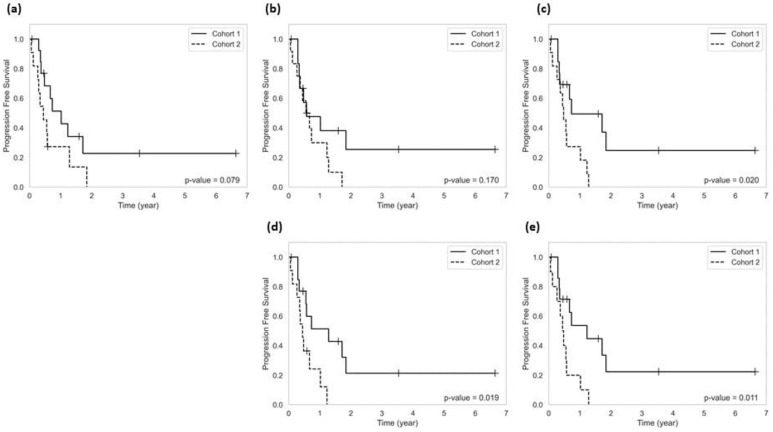
Kaplan–Meier progression-free survival curves for two cohorts of patients stratified based on the outcome prediction by the radiomic models developed with different segmentation modules: (**a**) Cascaded 2D UNets, (**b**) 3D UNets, (**c**) Cascaded 2D and 3D UNets, (**d**) Cascaded 2D and 3D UNets + MSGA, and (**e**) Ground-truth masks. Cohort 2 includes the patients in the independent test set who had at least one lesion with a predicted outcome of local failure, and cohort 1 includes all other patients in the independent test set.

Figure 4 demonstrates the results of survival analysis on the test set. The Kaplan–Meier progression-free survival curves are presented for two patient cohorts classified based on their predicted outcome by the radiomic models developed with different segmentation modules. A log-rank test applied on the survival curves of the two cohorts demonstrates no statistically significant difference for the models developed with cascaded 2D UNets or 3D UNet. However, a significant difference is observed between the survival curves of the cohorts stratified by the models developed with cascaded 2D and 3D UNets, cascaded 2D and 3D UNets + MSGA, and the ground-truth masks.

## 4. Discussion

Accurate segmentation of brain tumours on the baseline and follow-up images is an essential yet laborious task. It is required for therapy response assessment as well as for extracting imaging biomarkers in various diagnostic and prognostic applications. An automatic segmentation method streamlines the therapy outcome prediction and evaluation workflow (which requires ROI masks for analysis), potentially at the cost of less accuracy. In this study, we investigated the effect of tumour segmentation accuracy on efficacy of the MRI radiomic biomarkers extracted from the tumour and tumour margin for radiotherapy outcome prediction in BM. The results of our study show that while the impact of tumour delineation accuracy is considerable for less accurate segmentation models (dice score ≤ 0.85), the radiomic features and prediction models are relatively robust to imperfections in the generated tumour masks. In particular, the list of selected features associated with the ground-truth tumour masks and those generated using the cascaded 2D and 3D UNets + MSGA model (with an average dice score of about 0.90) shared six out of the seven features selected based on the mRMR method. Furthermore, the features extracted using the tumour masks generated by this segmentation model resulted in an outcome prediction model with fairly close performance metrics to those obtained by the ground-truth model, including the accuracy, sensitivity, specificity, AUC, and F1-score of 80% vs. 80%, 77% vs. 83%, 83% vs. 78%, 0.78 vs. 0.81, and 77% vs. 78%, respectively. Progression-free survival analyses demonstrated that both the outcome prediction models could stratify the patients into two cohorts (low-risk vs. high-risk) with statistically significantly different survival curves.

The effect of segmentation accuracy on the performance of radiomic features for different cancer sites and imaging modalities is a subject yet to be explored in the literature. Among research done so far, Jin et al. studied the effect of automatic segmentation using multiple UNet-based architectures on the accuracy of radiomic features for transvaginal ultrasound images of cervical cancer [33]. The results of that study show the feasibility and reliability of automatic segmentation, especially with UNet-based models, for relevant radiomic studies. This is in agreement with the observation of this study where fairly accurate tumour segmentation was found to be sufficient for radiomic models of cancer therapy response. Teng et al. studied the effect of automatic segmentation on preoperative lymph node status prediction models with radiomic features extracted from ultrasound for patients with early-stage cervical cancer. Their study shows that in some cases, automatic segmentation improves the prediction accuracy as human-derived segmentation methods introduce human bias into the radiomic process [34]. This is an interesting observation that may be valid only for smaller ROIs, such as lymph nodes, and/or low-SNR imaging modalities, such as ultrasound, but should be rigorously investigated in future works on large sample sizes for different cancer sites and imaging modalities.

The findings of this study show that radiomics in conjunction with machine learning can be used to predict radiotherapy outcome in brain metastasis early after treatment, where automatic tumour segmentation could potentially be utilized instead of manual segmentation to facilitate prediction model development and investigation. In clinic, these implications could be significant, as an early prediction of treatment response may lead to therapy adjustments which, in turn, enhance patients’ survival and quality of life. The observation of this study that radiotherapy outcome prediction in brain metastasis is not very sensitive to small inaccuracies in tumour segmentation permits high-throughput implementation and exploration of new computational prediction models to develop robust systems for clinical decision support.

## 5. Conclusions

The promising results of this study open the avenue to applying automatically generated segmentation masks for discovering diagnostic and prognostic biomarkers in BM without sacrificing their accuracy. This is a significant contribution considering the heavy burden that manual segmentation imposes on image-guided therapeutic systems in neuro-oncology, including the models for therapy outcome prediction and the platforms for therapy outcome evaluation. While the findings of this paper are promising and pave the way for future research, future investigations are required to further assess the conclusions of this study on a larger scale when imaging data are available from larger patient cohorts and possibly multicenter studies.

## Figures and Tables

**Table 1:** Dice similarity coefficient (DSC), Hausdorff distance (HD), and volume estimation error (VEE) for segmentation of brain metastasis using different network architectures. The best values in each column are shown in bold.

Segmentation Model		Baseline		First Follow-Up	
		Training Set	Test Set	Training Set Patients	Test Set Patients
Cascaded 2D UNets	DSC	0.85 ± 0.05	0.85 ± 0.06	0.82 ± 0.05	0.81 ± 0.07
	HD	3 ± 0.6 mm	3.4 ± 0.7 mm	3.46 ± 0.6 mm	3.7 ± 0.5 mm
	VEE	0.63 ± 0.44 cc 17.2% ± 6.1%	0.71 ± 0.47 cc 19.4% ± 8.3%	0.75 ± 0.5 cc 20.6% ± 8.5%	0.77 ± 0.51 cc 21.5% ± 9%
3D UNet	DSC	0.87 ± 0.06	0.85 ± 0.06	0.85 ± 0.05	0.82 ± 0.06
	HD	2.9 ± 0.8 mm	3.1 ± 0.82 mm	3.2 ± 0.85 mm	3.5 ± 0.6 mm
	VEE	0.6 ± 0.42 cc 15.8% ± 5.4%	0.7 ± 0.45 cc 17% ± 7%	0.72 ± 0.47 cc 18% ± 7.3%	0.75 ± 0.53 cc 18.9% ± 9.5%
Cascaded 2D and 3D UNets	DSC	0.89 ± 0.05	0.88 ± 0.05	0.86 ± 0.05	0.83 ± 0.05
	HD	2.45 ± 0.6 mm	2.65 ± 0.63 mm	2.8 ± 0.6 mm	3.1 ± 0.5 mm
	VEE	0.55 ± 0.35 cc 13.1% ± 4.2%	0.61 ± 0.4 cc 15.8% ± 6.5%	0.64 ± 0.43 cc 16.6% ± 6.8%	0.68 ± 0.5 cc 17.9% ± 7.7%
Cascaded 2D and 3D UNets + MSGA	DSC	**0.91 ± 0.03**	**0.90 ± 0.04**	**0.89 ± 0.04**	**0.87 ± 0.05**
	HD	**2.1 ± 0.45 mm**	**2.3 ± 0.55 mm**	**2.21 ± 0.5 mm**	**2.74 ± 0.49 mm**
	VEE	**0.42 ± 0.3 cc** **11.2% ± 3.9%**	**0.53 ± 0.36 cc** **12.8% ± 5.1%**	**0.57 ± 0.38 cc** **14.7% ± 4.7%**	**0.61 ± 0.48 cc** **15.9% ± 5.1%**

**Table 2:** List of the top seven selected features using the mRMR feature selection method for different segmentation models. Bold features are the shared ones with those obtained using the ground-truth segmentation masks.

Segmentation Model	Selected Features
Cascaded 2D UNets	wavelet-LLH_glcm_Correlation_T2_Margin
	wavelet-LHH_glrlm_RunVariance_T2
	original_gldm_DependenceVariance_T2_Margin
	wavelet-HLH_glcm_Imc2_T2_Margin
	wavelet-LHH_glcm_Idm_T2_Margin
	wavelet-LHL_gldm_SmallDependenceHighGrayLevelEmphasis_T1
	wavelet-HHH_glszm_ZonePercentage_T1_Margin
3D UNet	**wavelet-HLH_gldm_LargeDependenceLowGrayLevelEmphasis_T2**
	original_gldm_DependenceEntropy_T2_Margin
	wavelet-LHL_gldm_SmallDependenceHighGrayLevelEmphasis_T1
	wavelet-HLH_gldm_SmallDependenceLowGrayLevelEmphasis_T2
	wavelet-HHL_ngtdm_Contrast_T2
	wavelet-HLH_glcm_Imc2_T2_Margin
	original_gldm_DependenceVariance_T2_Margin
Cascaded 2D and 3D UNets	**wavelet-HHL_firstorder_Minimum_T1_Margin**
	**original_gldm_DependenceEntropy_T2_Margin**
	**original_glcm_Idn_T2_Margin**
	**wavelet-HLH_gldm_LargeDependenceLowGrayLevelEmphasis_T2**
	wavelet-LHL_glcm_Contrast_T1
	wavelet-HHH_gldm_DependenceVariance_T1_Margin
	wavelet-LHL_gldm_SmallDependenceHighGrayLevelEmphasis_T1
Cascaded 2D and 3D UNets + MSGA	**wavelet-HHL_firstorder_Minimum_T1_Margin**
	**original_gldm_DependenceEntropy_T2_Margin**
	**original_glcm_Idn_T2_Margin**
	**wavelet-HLH_gldm_LargeDependenceLowGrayLevelEmphasis_T2**
	**wavelet-LLL_ngtdm_Strength_T1_Margin**
	**wavelet-HLL_glcm_Idn_T1_Margin**
	wavelet-HHL_firstorder_Skewness_T1
Ground-Truth	wavelet-HHL_firstorder_Minimum_T1_Margin
	original_gldm_DependenceEntropy_T2_Margin
	original_glcm_Idn_T2_Margin
	wavelet-HLH_gldm_LargeDependenceLowGrayLevelEmphasis_T2
	wavelet-LLL_ngtdm_Strength_T1_Margin
	wavelet-HLL_glcm_Idn_T1_Margin
	wavelet-LHH_glszm_SizeZoneNonUniformityNormalized_T1_Margin

**Table 3:** Results of therapy outcome prediction using the radiomic models developed with different segmentation modules.

Segmentation Model	Independent Test Set				
	Accuracy	Sensitivity	Specificity	AUC	F1-Score
Cascaded 2D UNets	72.5%	70.6%	74%	0.62	68.5%
3D UNet	72.5%	70.6%	74.%	0.67	68.5%
Cascaded 2D and 3D UNets	77.5%	76.5%	78.2%	0.72	74.3%
Cascaded 2D and 3D UNets + MSGA	80%	76.5%	82.6%	0.78	76.5%
Ground-Truth	80%	82.5%	78.2%	0.81	77.8%

## Data Availability

Data were collected and available on request at the Odette Cancer Centre, Sunnybrook Health Sciences Centre, Toronto, ON, Canada, after institutional approval.
